# Comparative ligand structural analytics illustrated on variably glycosylated MUC1 antigen–antibody binding

**DOI:** 10.3762/bjoc.16.206

**Published:** 2020-10-13

**Authors:** Christopher B Barnett, Tharindu Senapathi, Kevin J Naidoo

**Affiliations:** 1 Scientific Computing Research Unit and Department of Chemistry, University of Cape Town, Rondebosch, 7701, South Africa; 2 Infectious Disease and Molecular Medicine, Faculty of Health Science, University of Cape Town, Rondebosch, 7701, South Africa

**Keywords:** binding, conformation, Galaxy, glycoprotein, in silico

## Abstract

When faced with the investigation of the preferential binding of a series of ligands against a known target, the solution is not always evident from single structure analysis. An ensemble of structures generated from computer simulations is valuable; however, visual analysis of the extensive structural data can be overwhelming. Rapid analysis of trajectory data, with tools available in the Galaxy platform, can be used to understand key features and compare differences that inform the preferential ligand structure that favors binding. We illustrate this informatics approach by investigating the in-silico binding of a peptide and glycopeptide epitope of the glycoprotein Mucin 1 (MUC1) binding with the antibody AR20.5. To study the binding, we performed molecular dynamics simulations using OpenMM and then used the Galaxy platform for data analysis. The same analysis tools are applied to each of the simulation trajectories and this process was streamlined by using Galaxy workflows. The conformations of the antigens were analyzed using root-mean-square deviation, end-to-end distance, Ramachandran plots, and hydrogen bonding analysis. Additionally, RMSF and clustering analysis were carried out. These analyses were used to rapidly assess key features of the system, interrogate the dynamic structure of the ligand, and determine the role of glycosylation on the conformational equilibrium. The glycopeptide conformations in solution change relative to the peptide; thus a partially pre-structuring is seen prior to binding. Although the bound conformation of peptide and glycopeptide is similar, the glycopeptide fluctuates less and resides in specific conformers for more extended periods. This structural analysis which gives a high-level view of the features in the system under observation, could be readily applied to other binding problems as part of a general strategy in drug design or mechanistic analysis.

## Introduction

A typical sequence of events in research and discovery is noticing a critical biological interaction, searching for structural data, and then searching for the molecular rationale. This is the connection between biology, chemical biology, and chemistry. The Galaxy project is a popular open web-based platform for accessible, reproducible, and transparent computational research [1]. Originally built to support bioinformatics, Galaxy now supports a much more expansive community including proteomics [2], metabolomics [3], cheminformatics [4], glycoinformatics [5], and chemistry [6]. Of value to these communities are the broad range of tools and ways to connect tools (workflows) in Galaxy that enable diverse, multidisciplinary research. In this paper, we show how an informatics approach provides a high-level overview, thus enabling rapid observations of changes in molecular details pertinent to the system under investigation. We apply this approach to the binding of glycosylated molecules for the well-known system of mucin binding to the AR20.5 murine antibody.

The binding of glycosylated biomolecules is of increasing interest as glycans are found to be involved in cellular functioning and messaging. The mucins, which are cell surface-associated glycoproteins, are found in mucous secretions and are heavily O-glycosylated [7]. Mucins serve several functions: including protecting the body from pathogens by forming chemical barriers and cellular signaling. Mucin 1 (MUC1) is tethered to the cellular membrane and is found to be aberrantly glycosylated and overexpressed in several epithelial cancers [8]. Further, it is thought to participate in the hyperactivation of selected intracellular signal transduction pathways that promote tumorigenicity [9]. MUC1 is a cancer biomarker that can be detected by serum biomarker assays (such as the CA15-3 test [10–11]). The mode of binding between MUC1 and antibodies has received much attention, and the specificity of this interaction is of interest in improving the performance of these biomarker assays [12–13].

The extracellular domain of MUC1 contains a variable number of tandem repeats (VNTR). The VNTR region is comprised of a repeating sequence of 20 amino acids (–His-Gly-Val-**Thr**-**Ser**-Ala-Pro-Asp-**Thr-**Arg-Pro-Ala-Pro-Gly-**Ser**-**Thr**-Ala-Pro-Pro-Ala–)*_n_*, and there are five sites where O-glycosylation may occur (indicated in bold). In cancerous cells, the glycans tend to be truncated or have additional sialylation [14]. For example, in mammary epithelial cells, the mixture of O-glycans that glycosylate mucins are *extended core* 2 structures, while in breast cancer cells, O-glycan mass decreases (hypoglycosylation), and there is an increase in abundance of *sialylated core* 1 [15]. The upregulation of Tn (αGalNAc) and STn (αNeuAc-2,6-αGalNAc) antigens are commonly associated with cancerous cells [14].

**Figure 1:**
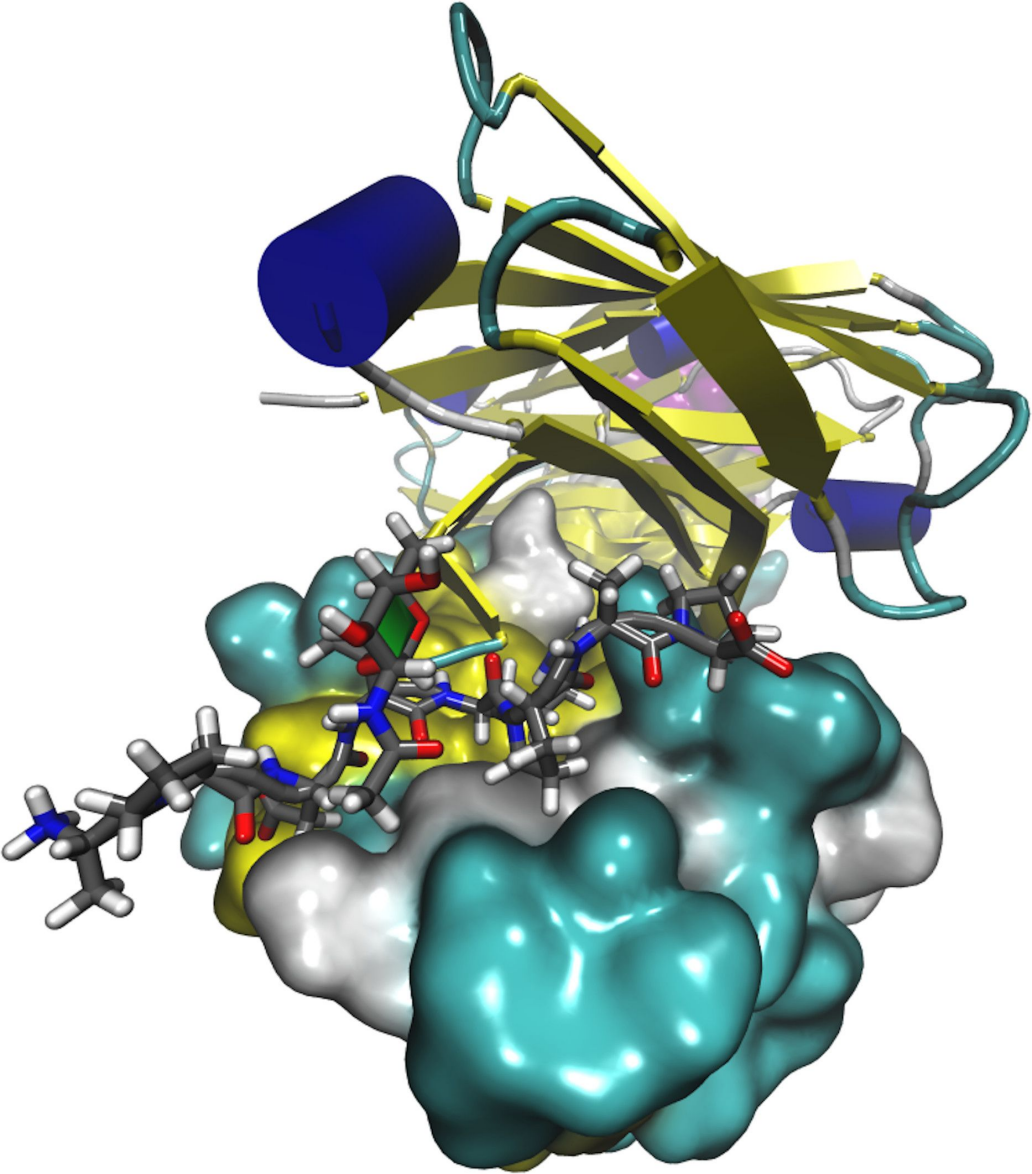
A representation of mucin glycopeptide bound to AR20.5 antibody. Chain A is represented as a molecular surface colored by secondary structure, chain B is represented in cartoon and colored by secondary structure. The mucin peptide is represented as licorice. The Tn glycan (*N*-acetylgalactosamine) is represented as licorice, and the sugar ring is highlighted with the paper chain representation [18–19].

Movahedin et al. confirmed that the glycosylation of MUC1 influences its binding to the AR20.5 murine antibody [16], specifically the Tn-antigen binds more strongly than the nonglycosylated antigen. AR20.5 is known to bind a specific epitope within the MUC1 VNTR domain. Thus, a synthetic 8-amino acid peptide (APDTRPAP) and the corresponding Tn glycopeptide were synthesized. It was found from the co-crystallization of the AR20.5 antigen-binding fragment (Fab) with the MUC1 peptide and glycopeptide that the glycan moiety of the glycopeptide did not bind to the antibody (Figure 1 and PDB ID:5T6P, 5T78). This is unusual considering that in previous experiments of murine antibody SM3 that Brooks [17] found the glycan forms part of the epitope and binds directly to the antibody. Movahedin et al. hypothesized that the glycan modulates the conformation of the peptide portion of the antigen and does not bind directly.

Previous studies have shown that O-glycosylation may provide increased physical stability [20], rigid conformations for protein stability [21], induce the formation of stiff and extended peptide conformations [22], and may affect peptide conformations near the glycosylation site and at distant sites [23]. In glycopeptide enkephalin analogs, the only observed conformational effects due to O-glycosylation were on the residue of attachment and its neighboring residue [24]. While for prion peptides, the O-glycosylation (α-GalNAc) is able to affect the structural transition and suppresses the formation of amyloid fibril formation [25]. The solution structure of O-glycosylated prion peptide was not shifted significantly, with only minor shifts seen in the vicinity of the glycosylation site. Yet there is a stabilization of the β-structure relative to the random coil and the effects of the glycosylation were hypothesized to relate to the conformational properties of the peptides in solution (as opposed to their equilibrium structures in solution) [25].

A comprehensive structural study of the O-glycosylation-induced changes in a mucin octapeptide showed that the peptide conformation depended on the extent of glycosylation. Glycosylation induces small changes in protein structure and shifts it from a random to a more turn-like structure [26]. Kirnasky et al. noted that O-glycosylation slightly affected the conformational equilibrium of the peptide backbone near the glycosylated residue for a 15-residue mucin peptide. The APDTRP fragment resembled an S-shaped bend and a clustering of low-energy conformations revealed structural similarities between glycosylated and nonglycosylated peptides [23].

The work by Movahedin et al. and others [14,16] provides a foundation for further investigation into the binding of glycopeptide antigens to antibodies using computational modeling. Molecular dynamics (MD) simulations and analysis thereof are a well-known ingredient of the in-silico process for mechanistic screening of glycopeptide fragment binding to antibodies. In this work, the peptide only antigen (Ala-Pro-Asp-Thr-Arg-Pro-Ala-Pro, APDTRPAP) and the Tn glycosylated antigen (APDT(Tn)RPAP) are considered in solution and complex with the AR20.5 antibody. The Tn-antigen is of interest as it is often found upregulated in breast cancer [11,13]. We use MD simulations to investigate the conformational behavior of (glyco)peptide antigens bound to the AR20.5 antibody and to investigate the hypothesis that the glycan modulates the conformation of the peptide portion of the antigen. Primarily showcasing a structural analytics approach, we aim to use the tools and workflows available as part of the Galaxy project to analyze MD simulations to find out if the sugar moiety of the Tn-antigen binds directly to the antibody. Further, if the sugar does not bind directly (as found previously), then we will use these analyses to observe how the sugar modulates binding.

## Methods

The inputs, simulation scripts, Galaxy workflows (a series of tools and dataset actions that run in sequence), and data for these simulations are available at https://github.com/chrisbarnettster/bjoc-paper-2020-sm.

### Simulation

There is an increasing number of software available to assist with the building up of glycosylated biomolecular systems. As opposed to manual preparation, there are glycan-specific tools and toolkits such as doGlycans [27], Glycosylator [28], and online platforms such as GLYCAM-WEB [29] and CHARMM-GUI [30]. In this work, the CHARMM-GUI server [30] which includes several helper tools (PDB Manipulator [31] and Glycan Reader [32–33]), was used to build these systems and generate input files [34] for use with OpenMM.

Five systems were built in CHARMM-GUI based on initial structures from the Protein Data bank (PDB ID:5T6P, 5T78). The assumption was made that the Tn-antigen binds as per the PDB structure, and other modes of binding are not possible. The solvated receptor, solvated antigens (both the nonglycosylated and Tn-antigen), and a solvated complex (with both antigens) were built in 0.15 M KCl aqueous solution at 310.15 K (physiological temperature). Missing amino acid residues were added. Energy minimization and MD (equilibration and production) simulations were performed using OpenMM [35] and the CHARMM36 force field [36] using the OpenCL platform with mixed precision. Equilibration and production dynamics were carried out as per the scripts provided with CHARMM-GUI, except for adjustments to the time step and number of iterations. The calculations were carried out using Nvidia V100 GPUs.

The equilibration step included 5000 steps of minimization follows by 25000 steps of NVT dynamics (constant volume and temperature) with a time step of 0.001 ps. The particle mesh Ewald (PME) method was used. Nonbonded interactions were cut-off using the force-switching method from 10 Å to 12 Å, and hydrogen bonding constraints applied. During equilibration, the protein backbone and side chains were restrained (force constants of 400.0 and 40.0 kJ mol^−1^ nm^−2^ were used, respectively). The production dynamics were simulated using an *NpT* ensemble and using a time step of 0.002 ps. The antigen–antibody complex in solution was run for 210 ns, while the antigen was run for 500 ns. The antibody was run for 100 ns.

### Analysis

The majority of the analyses was carried out using Galaxy, the popular open web-based platform for bioinformatics and computational data analysis, which enables the creation of repeatable analysis pipelines (workflows). There are several well-known molecular dynamics analysis packages (MDAnalysis [37], Bio3D [38] and MDTraj [39]) which are available as computational chemistry analysis tools in Galaxy [6], and these were used to analyze the molecular dynamics trajectories.

**Figure 2:**
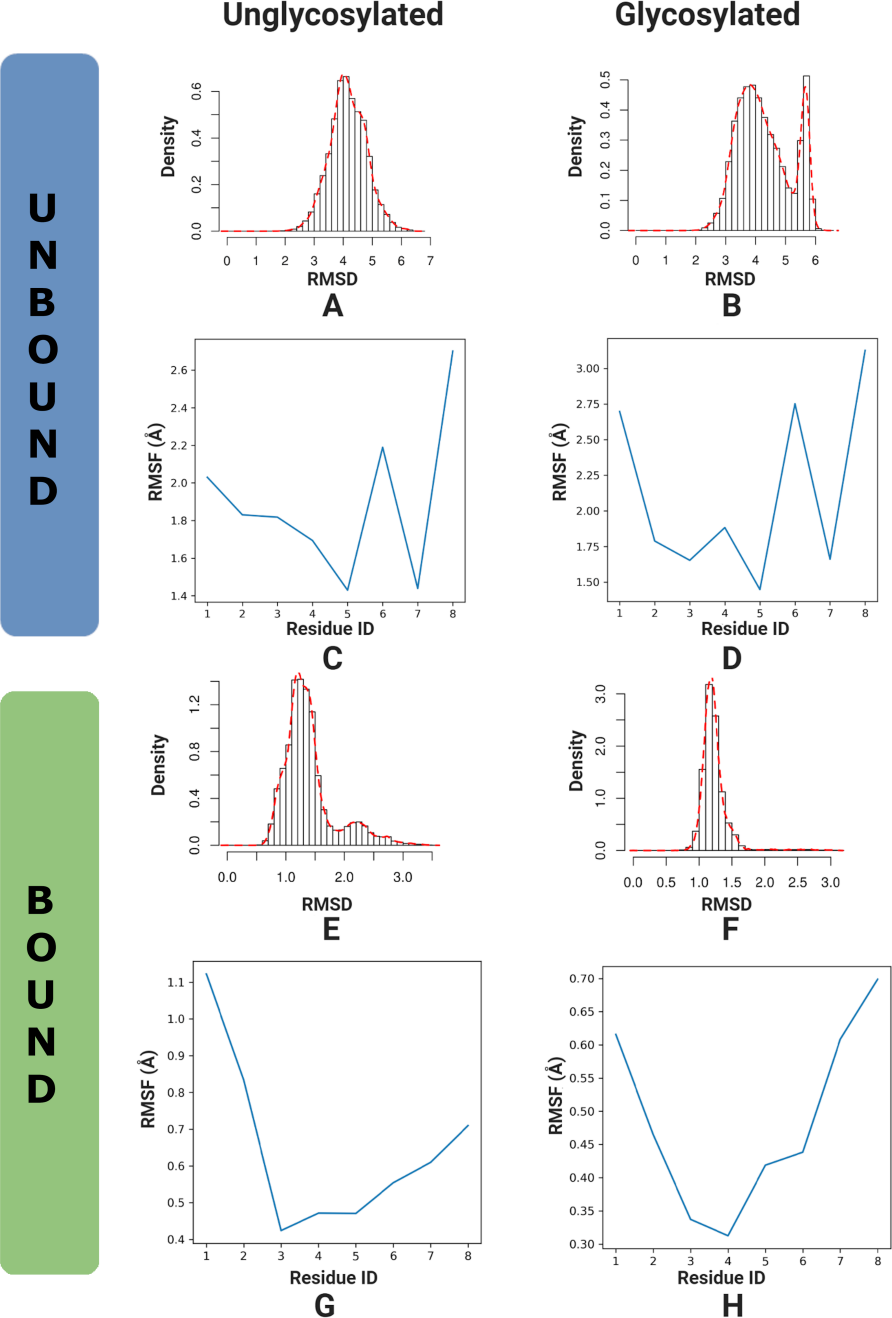
A comparison of root mean analyses for the antigen and Tn-antigen in solution (unbound) and in antibody (bound). RMSD histograms in solution (A, B) and antibody (E, F). RMSF’s in solution (C, D) and antibody (G, H). The graphs on the left are for the antigen and those in the right panel are for the Tn-antigen.

The root-mean-square deviation (RMSD) is calculated to measure the stability and conformation of a set of selected atoms. The RMSD is a standard measure of the structural distance between coordinate sets that measures the average distance between a group of atoms [40]. The peptide portion of the antigens was selected for analysis. The root-mean-square-fluctuation (RMSF) represents the deviation at a reference position over time and was calculated in order to measure the variability of the carbon backbone (C-α atoms were selected) of the peptide portion of the antigen (Figure 2).

**Figure 3:**
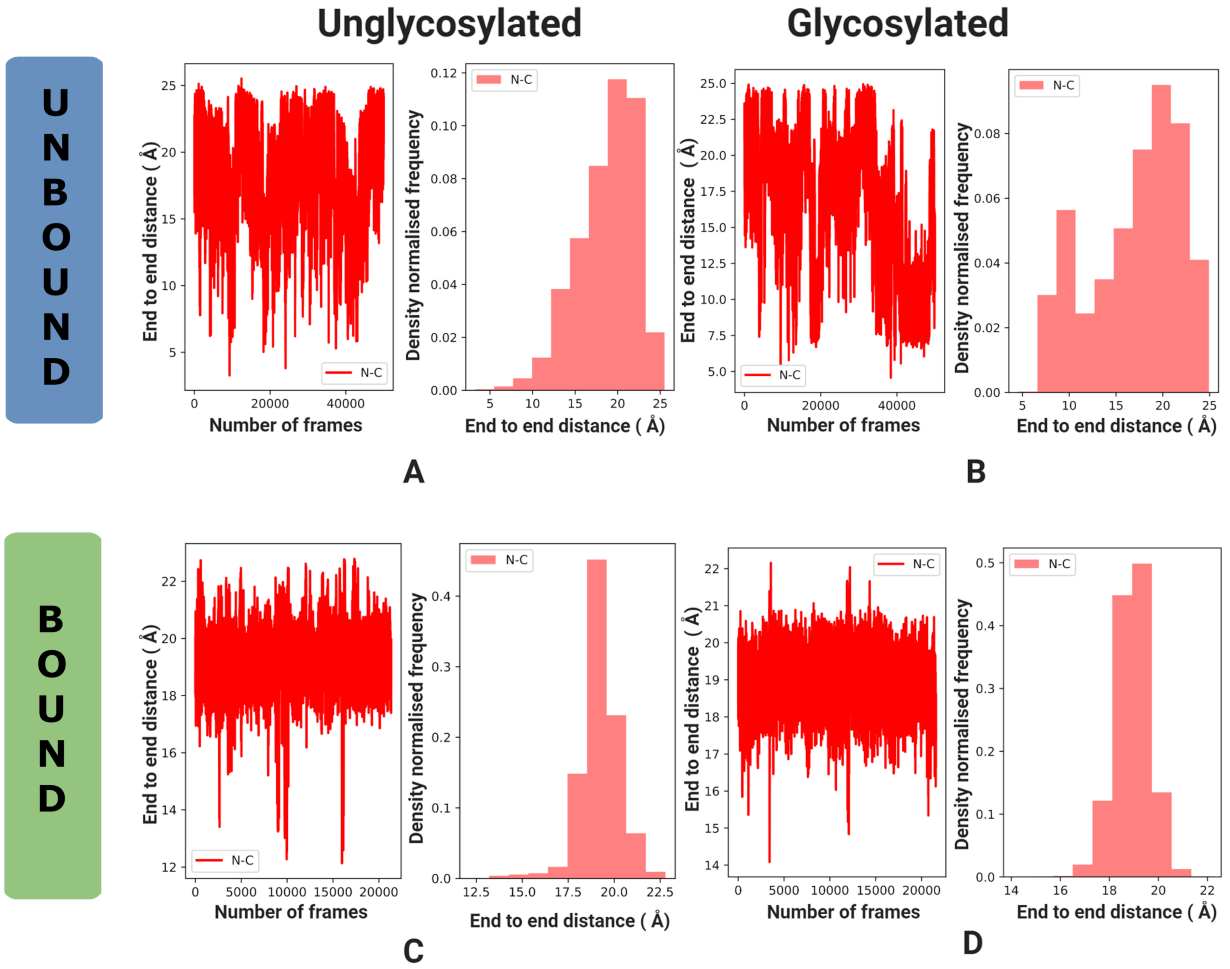
End-to-end time series and histogram for the antigen and Tn-antigen in solution (A, B) and the antibody (C, D). Plots for the antigen are in (A, C) and for the Tn-antigen in (B, D).

The end-to-end distance (displacement length) was used as a metric to understand the mobility and conformation of the peptide portion of the antigen throughout the simulation. This is defined as the carbon–nitrogen distance between the first and last amino acid residues of the antigen. A time-series analysis provides some insight, while a histogram provides a clearer understanding of the most populated conformations (Figure 3).

**Figure 4:**
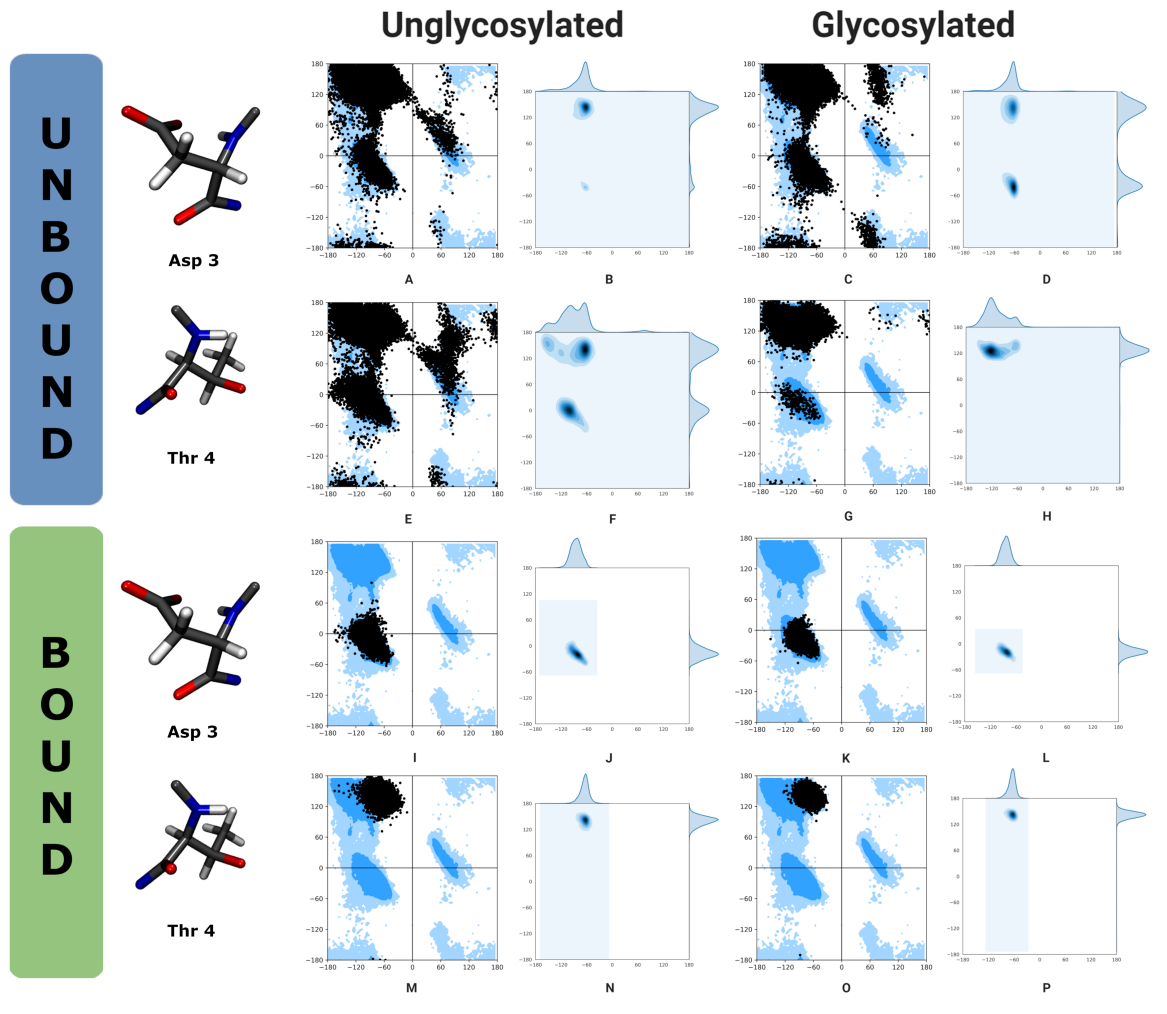
A comparison of Ramachandran analyses for two key amino acids, Asp3 and Thr4. The first row (A–D) illustrates the φ–ψ angles for amino acid 3 of the peptide, aspartate, with a scatter plot showing the allowed φ–ψ regions highlighted in blue (A), and a probability density Ramachandran plot (B) for the antigen, and a scatter plot (C) and probability density Ramachandran plot (D) for the Tn-antigen. While the second row (E–H) illustrates the φ–ψ angles for amino acid 4 of the peptide, threonine. The left panel of Ramachandran plots are for the antigen, and the right panel are for the Tn-antigen. The first two rows are for the antigens in solution (unbound, A–H), while the final two rows are for the antigens bound to the antibody (I–P).

A Ramachandran plot [41] is a well-known method for investigating the φ–ψ (phi–psi dihedral angle) preferences around protein backbones (Figure 4). All φ–ψ angles for the peptide portion of the antigens were measured for each frame of the simulation and aggregated per residue. The glycosidic-linkage dihedral angles of the Tn-antigen (in solution and bound to antibody) were also measured. A standard hydrogen-bonding analysis using MDAnalysis and VMD was carried out with the default angle cut-off and distance cut-off.

**Figure 5:**
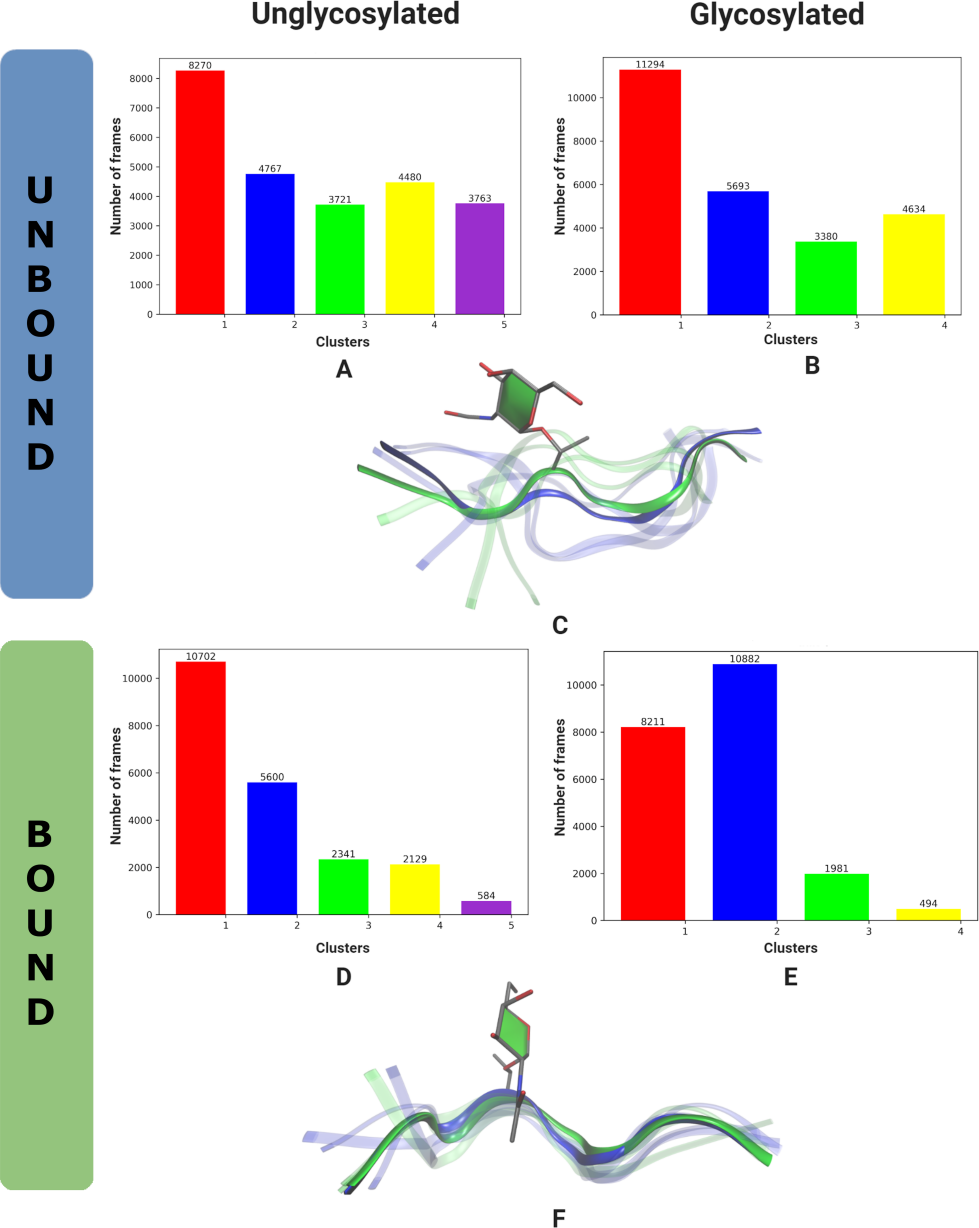
Distribution of clusters, found using TTClust, for the antigen and Tn-antigen in solution (A, B) and when bound to the antibody (D, E). The conformation of the clusters for solution (C) and bound (F) where the antigen is drawn as a blue ribbon with first cluster conformation in opaque blue. The Tn-antigen is drawn as a green ribbon with first cluster conformation in opaque green. The sugar is drawn without hydrogens in licorice and the sugar ring is highlighted with the paper chain representation [18–19].

A cluster analysis of the peptide portion of the antigen was carried out (Figure 5) using TTClust [42]. The clusters were chosen automatically based on the carbon backbone of the peptide portion of the antigen and clustered using the Ward algorithm.

## Results

The antigens were simulated in solution to understand the innate flexibility prior to binding to the antibody, and then also simulated in the complex with AR20.5 MUC1 antibody to understand the effect of glycosylation on antigen conformation during binding. With the rationale that a high-level overview can be used to understand the molecular changes, various analyses were considered: root-mean-square, end-to-end distance, clustering, φ–ψ backbone dihedral angles, and hydrogen-bonding interactions. These analyses focused primarily on the antigen as the antibody conformation does not change significantly in the time frame of the simulation. The peptide-only antigen will be referred to as the ‘antigen’ while the Tn-glycosylated antigen will be referred to as the ‘Tn-antigen’.

### Root-mean-square-analysis

In solution (unbound), the RMSD (Figure 2) has a broad spread and a similar center for both the antigen and Tn-antigen. It is readily apparent that the glycosylated antigen has a bimodal distribution (secondary peak at 5.7 Å), indicating at least one other interesting conformation. On consideration of the RMSD for the bound antigens, a narrowing in the distributions is noted. Bound Tn-antigen (Figure 2F) has the narrowest distribution, with a spread from 0.8 Å to 1.6 Å; this unimodal distribution is centered at 1.25 Å. There is no longer a secondary peak, indicating that there is restricted movement on binding. The bound antigen (Figure 2E), instead has a bimodal distribution with a significant population centered at 1.25 Å, a minor population centered at 2.25 Å, and a broad tail that extends to 3.5 Å. While there is restricted movement on binding, the antigen shows unexpected flexibility and a secondary peak at 2.25 Å. From the RMSD, we can infer there is a much tighter range of structures for both antigens when bound than in solution (this should be apparent as there is restricted motion due to the binding of the antigen to the antibody) and the bound Tn-antigen has a more defined and stable conformation.

The RMSFs of the two antigens in solution (Figure 2C and D) have a similar trend with fluctuations ranging between 1.4 Å and 3 Å. Both have large fluctuations, especially for Ala1, Pro6, and Pro8. The Tn-antigen RMSF fluctuates more than the antigen especially for Ala1, Thr4, and Pro8, respectively. When bound, both antigens show restricted fluctuations (Figure 2G and H), with the Tn-antigen showing less fluctuation about each residue. The first and last residues still fluctuate but all RMSF values are less than 1.1 Å indicating relatively minor fluctuations occur for the C-α carbons of the peptide backbone. Another noticeable change is the shift in Pro6, which fluctuated significantly in solution, and now does not. The antigen fluctuates most at the first residue, Ala1, and least at Asp3 and Thr4, while the Tn-antigen fluctuates most for the first and last residues, Ala1 and Pro8, and least for Asp3 and Thr4.

### End-to-end analysis

In solution (Figure 3A and B), the displacement lengths of the antigens have a similar range (3.0 Å to 25.0 Å vs. 6.5 Å to 25.0 Å), and both antigens adopt a wide range of conformations with a preference for extended structures. There is a tendency for the Tn-antigen to also prefer a compact conformation, as per the sampling seen at 9.5 Å in the histogram (Figure 3B). The antigen has a left-skewed distribution centered at 19.5 Å, while the Tn-antigen could be bimodal (see the sampling at 9.5 Å and 19.5 Å) or a left-skewed unimodal distribution centered at 19.5 Å.

In contrast, the bound antigens have a much narrower spread (Figure 3C and D). The end-to-end distance for the antigen ranges from 12.5 Å to 22.5 Å, with a distribution centered at 18.9 Å; while the Tn-antigen end-to-end distance ranges from 16.0 Å to 22.0 Å and is centered at 19.5 Å. This is a short peptide so the head and tail regions do fluctuate which could explain the significant spread in the end-to-end distance even though the antigen is bound to the antibody. Nevertheless, the Tn-antigen shows a slightly narrower spread and a more compact ensemble of structures, but otherwise, the end-to-end distance is very similar for both bound antigens.

### Ramachandran analysis

The φ–ψ angles of the antigens are considered using a Ramachandran plot. Figure 4 shows a Ramachandran plot for two key amino acids, the glycosylated threonine (Thr4) and neighboring aspartate (Asp3), and considers the φ–ψ angles over all frames of the simulation grouped for these residues. Detailed Ramachandran plots are available (Figures S1 and S2 in Supporting Information File 1) for all residues that can be measured (residues 2–8).

Ramachandran plots show that the φ–ψ distribution for the antigens differs in solution but is the same when bound to the antibody. This is a prestructuring effect and is likely an important contributor to the improved binding affinities seen for the Tn-antigen.

In solution, the third residue (Asp3) prefers (−60°, 135°) for the antigen (Figure 4A and B) with some sampling at (−60°, −40°) and minimal sampling at (60°, 60°). When glycosylated, the ψ sampling shifts to become a balanced bimodal distribution (Figure 4C and D) with similar sampling at (−60°, 135°) and (−60°, −40°), and minimal sampling seen at (60°, 160°) and (60°, −170°). Note that the probability distribution gives the best indication of relevant regions. The fourth residue (Thr4) shows multimodal sampling in φ and a bimodal distribution in ψ, with conformers at (−100°, 0°) and (−60°, 130°) being preferred for the antigen (Figure 4E and F). However, when glycosylated the sampling of Thr is restricted (Figure 4G and H), with a strong preference for (−120°, 120°) and the ψ distribution is effectively unimodal.

The antibody prefers that both antigens adopt a particular shape to fit, and this is seen in the φ–ψ distributions, which shift for Asp3 and Thr4. When bound, both antigens have an almost identical φ–ψ distribution except that the peaks are slightly narrower for the Tn-antigen. In some cases, the preference stays the same and reduced flexibility is observed, for example, Pro2 (Figure S1 and S2 in Supporting Information File 1). In other cases, the conformational preferences shift on binding but this shows no correlation to the effect of glycosylation, for example, Pro6, Ala7 (Figure S1 and S2 in Supporting Information File 1), and finally, the conformational preference seen for glycosylation in solution aligns with the preference seen for both bound antigens, for example, Asp3 and Thr4 (Figure 4I–P).

For Asp3, the φ–ψ preference for both bound antigens is (−60°, −40°), which correlates with the shift seen on glycosylation in solution where the φ–ψ preference moved from (−60°, 135°) to sample an additional region of phase space and a combination of conformations at (−60°, −40°) and (−60°, 135°). For Thr4, the φ–ψ preference for both bound antigens is (−65°, 140°) which correlates with the shift seen on glycosylation in solution where the φ–ψ preference moved from (−100°, 0°) and (−60°, 130°) to (−120°, 120°). The antibody binds both glycosylated and unglycosylated antigen with the same conformational preference at Asp3 and Thr4 which correlates with the preferred states seen for the glycosylated antigen in solution. There is some evidence of a pre-structuring or pre-organization effect, where O-glycosylation shifts the conformational equilibrium of the peptide towards conformations that are pre-organized for antibody binding.

A Ramachandran plot can be used to understand the role of the sugar moiety, by comparison of the dihedral angle distribution of the glycosidic linkage between the glycan and peptide portion of the Tn-antigen (Figure S3 in Supporting Information File 1). In solution, there is a preference for (70°, 100°) with limited sampling observed in the negative regions of the ψ distribution. On binding, this preference is limited and changes slightly to (70°, 120°) with no sampling observed in the negative regions of the φ distribution.

### Cluster analysis

A cluster analysis of the solution structures yields 5 clusters for the antigen and 4 clusters for the Tn-antigen (Figure 5A and B). The predominant conformer in both antigens is the extended form (Figure 5C), while for the Tn-antigen, the fourth cluster exhibits a more compact conformation (a transparent green conformer in Figure 5C) as noted in previous analysis.

A cluster analysis of the bound antigens yields 5 clusters for the antigen and 4 clusters for the Tn-antigen (Figure 5D and E). The predominant conformer in both antigens is similar (Figure 5F), as noted in previous analysis. For the antigen, the first cluster dominates (43%) with the second cluster about half as many members (22%), and the third cluster accounting for 9% of all conformations analyzed. For the Tn-antigen, the first and second clusters dominate accounting for respectively 33% and 44% of all conformations analyzed.

The cluster analysis indicates key conformations of the antigens seen in solution and when bound. In solution, the Tn-antigen can adopt a compact conformation while both antigens adopt extended structures when bound to the antibody. When considering the population count (Figure 5D and E) and residence time of the clusters (Figures S4 and S5 in Supporting Information File 1), the bound Tn-antigen is able to stay resident in the dominant conformation without regularly flipping to other conformations.

### Hydrogen bonding

The specifics of intermolecular interactions can also be considered, and here we utilized a hydrogen-bonding analysis to consider how the sugar moiety could interact with the antibody (Tables S1–S7 in Supporting Information File 1).

In solution, hydrogen bonds occur within the antigen between Arg5–Asp3 and Arg5–Pro8 (in order donor–acceptor) with occupancies of 31.83% and 14.32% (and 13.67%). For the Tn-antigen, the peptide portion has hydrogen bonds between Arg5–Pro8 (26.69% and 26.58%), Arg5–Asp3 (12.45%), an Arg5–Pro2 interaction is observed with an occupancy of 7.13%, and an intramolecular hydrogen bond between the C3 alcohol and the carbonyl of the *N*-acetyl moiety of the GalNAc has an occupancy of 6.92%. A shift in hydrogen-bonding populations on glycosylation and the appearance of the Arg5–Pro2 (7.13%) interaction aligns with the compact structure noted previously for the Tn-antigen.

When bound, additional intramolecular hydrogen bonds are observed for the Tn-antigen with interactions between the GalNAc–Thr4 (NH of the acetyl group to carbonyl group) and GalNAc–GalNAc (NH of the acetyl group and the C3 alcohol with the carbonyl of the *N*-acetyl moiety), which occur with occupancies of 23.04% and 29.08%, respectively. These two hydrogen bonds may play a crucial role in maintaining the conformation of the Tn-antigen. There are no intramolecular hydrogen bonds between the peptide moiety of the antigens; these are replaced by hydrogen-bonding between the antigen and chain A of the antibody. The following hydrogen bonds occur between the antigen and antibody: Arg5–Glu39 (141.21%, above 100% as counting both acceptor sites on Arg), Lys58–Asp3 (44.44%), Tyr37–Pro2 (42.55%), Arg55–Asp3 (38.11%), and Tyr54–Asp3 (28.51%). The following hydrogen bonds occur between the Tn-antigen and chain A of the antibody: Arg5–Glu39 (137.49%, above 100% as counting both acceptor sites on Arg), Lys58–Asp3 (42.80%), Tyr37–Pro2 (46.73%), Arg55–Asp3 (37.77%), and Tyr54–Asp3 (31.44%). A hydrogen bond (0.15%) was observed between the hydroxy group of Tyr100 of chain B of the antibody and the 6-hydroxy group of the GalNAc. While seemingly short-lived, it occurs with some frequency throughout the simulation (see Figure S6 in Supporting Information File 1). Movahedin et al. hypothesized that the glycan modulates the conformation of the peptide portion of the Tn-antigen and does not bind directly, noting that in the crystal structure GalNAc is positioned 4 Å away from the side chain of Tyr100, and indicating that any dispersion interactions would be insufficient to explain a 20-fold increase in affinity. It is unlikely that this hydrogen bond explains a 20-fold increase in affinity yet note that the mobility of the glycan moiety allows the hydrogen-bond interaction to occur. The hydrogen-bonding preferences and occupancies between the antigens and the antibody are very similar.

## Discussion

RMSD, RMSF, end-to-end distance, and Ramachandran analyses support that the Tn-antigen has slightly less conformational play than the nonglycosylated antigen when bound to the antibody. The analysis of the φ–ψ preference showed that the antibody binds both glycosylated and unglycosylated antigen with the same conformational preference (at Asp3 and Thr4) as that of the glycosylated antigen in solution. There is some evidence of a prestructuring or preorganization effect, where O-glycosylation shifts the conformational equilibrium of the peptide towards conformations that are preorganized for antibody binding. This should decrease the overall entropic penalty upon binding, and therefore would explain an increased binding affinity for the glycosylated antigen.

A cluster analysis showed that the dominant conformation for the bound antigens are similar. Intramolecular hydrogen-bonding interactions within GalNAc were more dominant in the antibody (have a higher occupation) than in solution. An intramolecular hydrogen bond within the Tn-antigen between the GalNAc–Thr4 (NH of the acetyl group to carbonyl group) may be responsible for maintaining the conformation of the Tn-antigen. The role of the sugar in excluding water was not investigated. A short-lived intermolecular hydrogen bond (0.15%) was observed between Tyr100 and GalNAc, and this is unlikely to be significant. These results correlated with the hypothesis put forward previously that glycosylation alters the conformational equilibrium of the antigen.

## Conclusion

We have shown how an informatics approach can be used to rapidly obtain key indicators of structural features for understanding the molecular level behavior of a system. We illustrated this informatics approach for the binding of glycosylated molecules, in particular for variably glycosylated mucin in solution and when bound to an antibody. RMSD, end-to-end distance, Ramachandran analysis, and hydrogen-bonding analyses were carried out using the Galaxy platform. Additionally, RMSF and cluster analysis were carried out. These analyses were used to gain rapid insight into the behavior of the system. The solution conformations of the Tn-antigen and the antigen were generally extended, yet the Tn-antigen was found to sample a more compact conformation. When bound to the antibody, both antigens had considerably less freedom than when in solution, as expected, and the Tn-antigen had less conformational play. However, this was not the result of hydrogen-bonding interactions between the glycan and the antibody or significantly different interactions between the peptide portion of the Tn-antigen and the antibody. Instead, contributing factors included an intramolecular hydrogen-bonding interaction between GalNAc and Thr4, and a preorganization effect (seen from Ramachandran analysis), where O-glycosylation shifted the conformational equilibrium of the peptide towards conformations that are preorganized for antibody binding. The results agreed with previous findings that glycosylation may affect peptide conformations near the glycosylation site and correlated with the hypothesis that glycosylation alters the conformational equilibrium of the antigen. This structural analysis which gives a high-level view of the features in the system under observation, could be readily applied to other binding problems as part of a general strategy in drug design or mechanistic analysis.

## Supporting Information

File 1 Additional molecular dynamics analyses.
