# Supplementary material for: Comparative ligand structural analytics illustrated on variably glycosylated MUC1 antigen–antibody binding
Source: Beilstein J Org Chem. 2020 Oct 13;16:2540–50. doi: 10.3762/bjoc.16.206 (PMC7590620; doi:10.3762/bjoc.16.206)
Supplement: File 1 — Additional molecular dynamics analyses. [file Beilstein_J_Org_Chem-16-2540-s001.pdf]

BEILSTEIN JOURNAL OF ORGANIC CHEMISTRY

Supporting Information

for

Comparative ligand structural analytics illustrated on variably glycosylated MUC1 antigen–antibody binding

Christopher B. Barnett, Tharindu Senapathi and Kevin J. Naidoo

Beilstein J. Org. Chem. **2020,** *16,* 2540–2550. doi:10.3762/bjoc.16.206

Additional molecular dynamics analyses

License and Terms: This is a supporting information file under the terms of the Creative Commons Attribution License (https://creativecommons.org/licenses/by/4.0). Please note that the reuse, redistribution and reproduction in particular requires that the authors and source are credited.
The license is subject to the *Beilstein Journal of Organic Chemistry* terms and conditions: (https://www.beilstein-journals.org/bjoc)

The inputs, workflows and data for these simulations are available at https://github.com/chrisbarnettster/bjoc-paper-2020-sm.

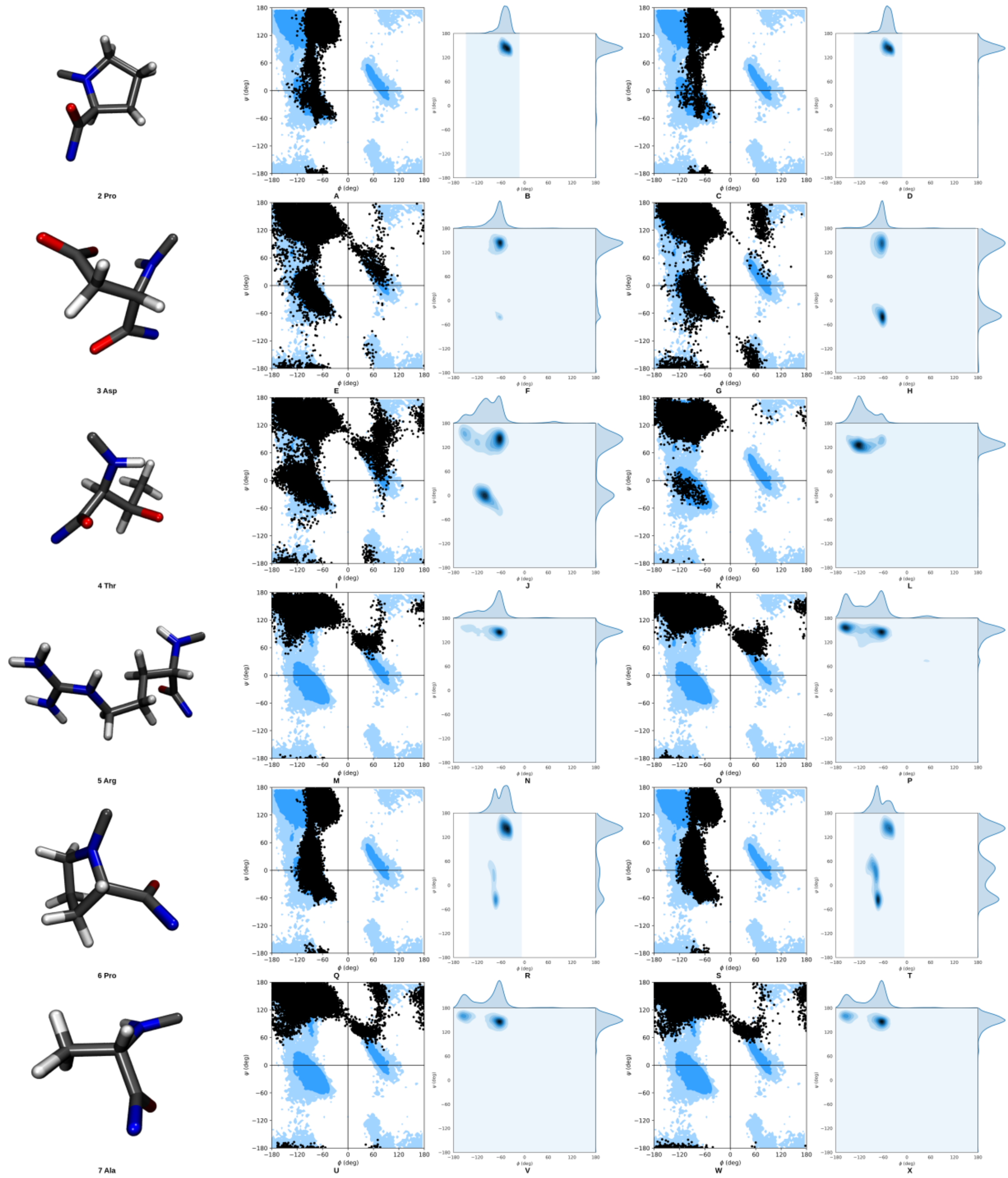

Figure S1: A comparison of all Ramachandran analyses for all residues of the antigen (left column, A, B) and Tn-antigen (right column, C, D) in solution. The first row illustrates the φ–ψ angles for amino acid 2 of the peptide, proline, with a scatter plot showing the allowed φ–ψ regions highlighted in blue (A), and a probability density Ramachandran plot (B) for the antigen, and a scatter plot (C) and probability density Ramachandran plot (D) for the Tn-antigen. The last row is for amino acid 7, alanine. By definition, the φ–ψ of residue 1 and residue 8 cannot be calculated.

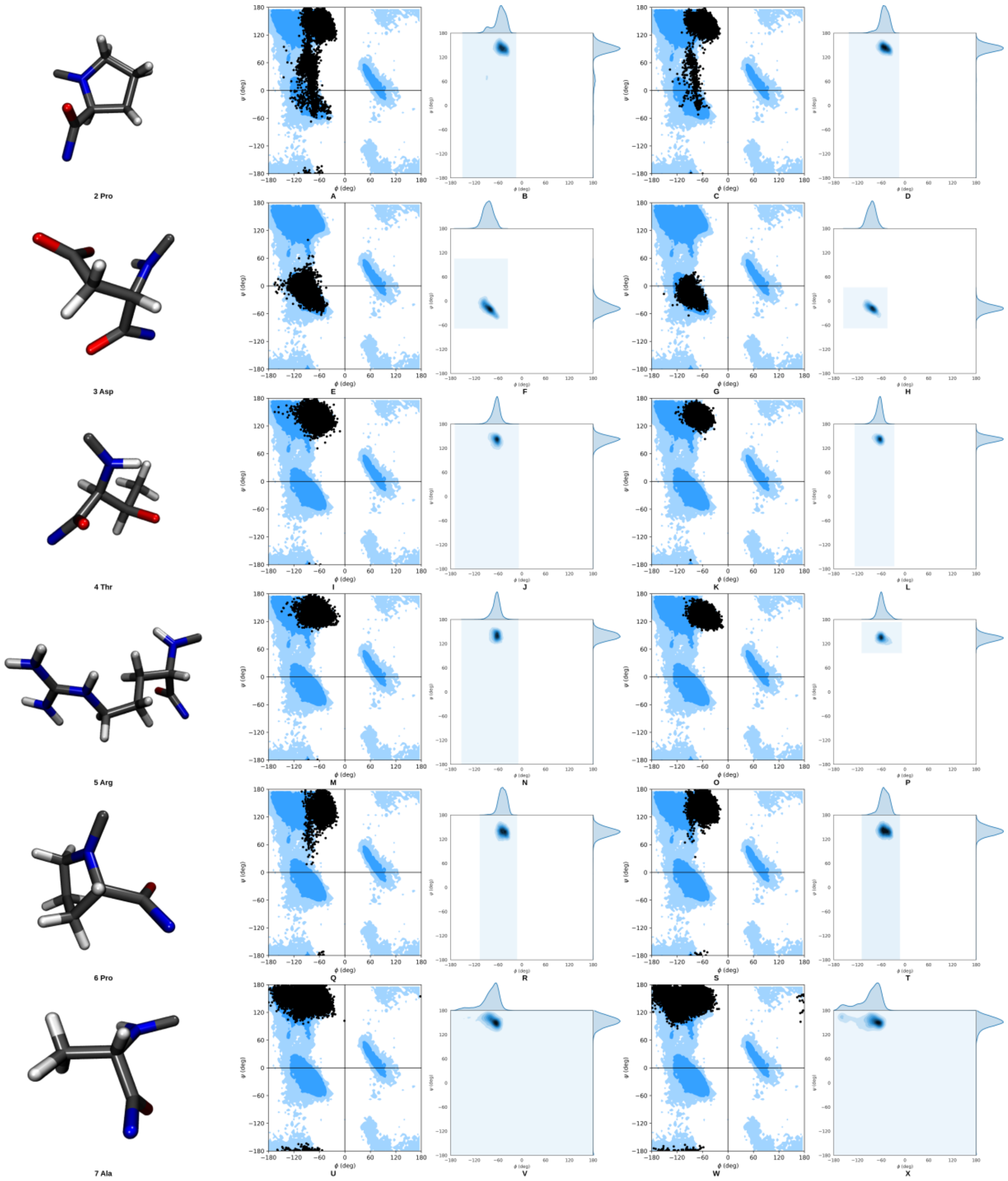

Figure S2: A comparison of Ramachandran analyses for the antigen (left column, A, B) and Tn-antigen (right column, C, D) bound to the antibody. The first row illustrates the φ–ψ angles for amino acid 2 of the peptide, proline, with a scatter plot showing the allowed φ–ψ regions highlighted in blue (A), and a probability density Ramachandran plot (B) for the antigen and a scatter plot (C) and probability density Ramachandran plot (D) for the Tn-antigen. The last row is for amino acid 7, alanine. By definition, the φ–ψ of residue 1 and residue 8 cannot be calculated.

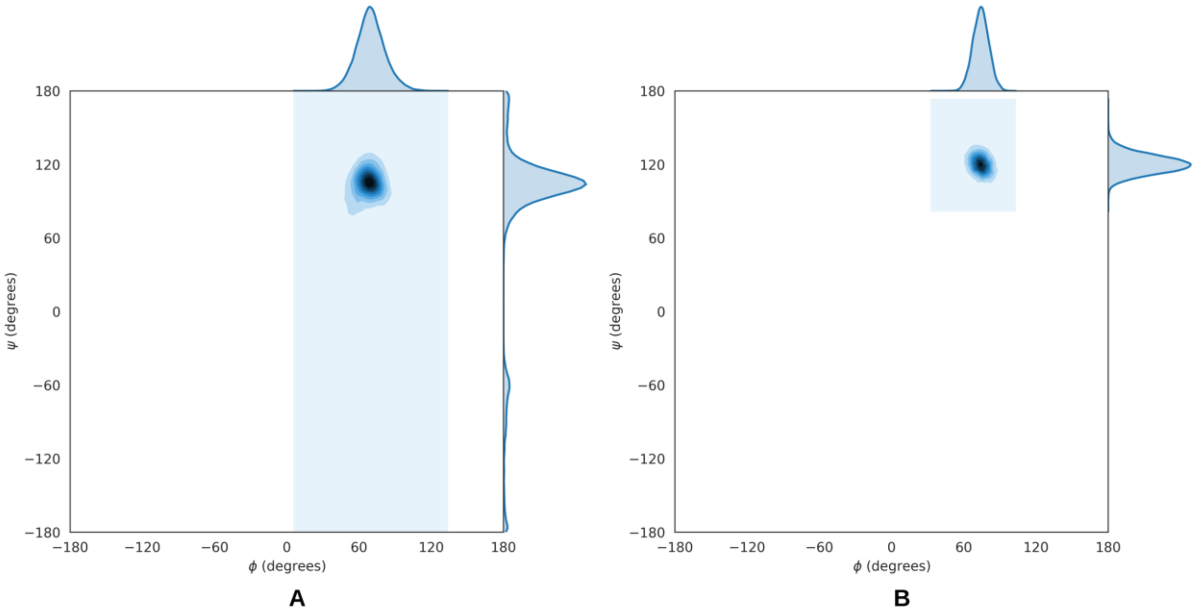

Figure S3: Ramachandran plot of the glycopeptide linkage of the Tn-antigen in water (A) and Ramachandran plot of the glycopeptide linkage of the Tn-antigen in complex with AR20.5 (B)

Ramachandran analysis for antigens in solution

For the second residue (Pro2), the preference is for (−50°,135°). Figure S1 A shows a scatter plot of the sampled φ–ψ angle, while in Figure S1 B this data is binned to generate a probability distribution. On glycosylation, this preference stays the same for both antigens. For the third residue (Asp3), (−60°,135°) is preferred for the antigen with some sampling at (−60°, −40°). While in the Tn-antigen, the ψ sampling becomes a balanced bimodal distribution with similar sampling at (−60°,135°) and (−60°, −40°).

For Thr4, φ sampling is multimodal in φ and bimodal in ψ, with conformers at (−100°, 0°) and (−60°, 130°) being preferred for the antigen. However, when glycosylated, the sampling of Thr is restricted, with a strong preference for (−120°, 120°) and the ψ distribution is effectively unimodal. For Arg5, the most sampled region is at (−60°, 150°) and when glycosylated, this resolves into two peaks at (−60°, 150°) and (−150°, 150°). Some minor sampling is noted in the (60°, 60°) region.

For Pro6, the most sampled region is (−60°, 150°) with (−90°, −30°) and (−90°, 30°) also sampled. When glycosylated, there is a shift in preference towards the negative in φ and a shift towards the negative in ψ, with the (−90°, −30°) conformer dominating but otherwise almost equal sampling of these conformations in ψ (see trimodal distribution). For Ala7, (−60°, 150°) and (−150°, 150°) are sampled with a preference for (−60°, 150°). On glycosylation, similar behavior is observed.

Ramachandran analysis for antigens bound to the antibody

The Ramachandran plots for each amino acid (Figure S2) show unimodal distributions with a single preference in φ and ψ. The second residue and seventh residues are exceptions. Pro minimally samples the negative ψ region, and this is observed less for the Tn-antigen, while Ala explores the top left of the β region (−160,160). The specific preferences for the φ–ψ distribution of the antigen, when bound to the peptide, can be compared to solution distributions (compare Figure S1 and S2). In some cases, the preference stays the same and reduced flexibility is observed, for example, Pro2. In other cases, the conformational preferences shift on binding but this shows no correlation to the effect of glycosylation, for example, Pro6, Ala7, and finally, the conformational preference seen for glycosylation in solution aligns with the preference seen for both bound antigens, for example, Asp3 and Thr4.

For Asp3, where the φ–ψ preference for both bound antigens is (−60°, −40°). In solution, glycosylation shifted the φ–ψ preference from (−60°,135°) to sampling an additional region of phase space and a combination of conformations at (−60°, −40°) and (v60°,135°). For Thr4, the φ–ψ preference for both bound antigens is (−65°, 140°). In solution, glycosylation shifted the φ–ψ preference from (−100°, 0°) and (−60°, 130°) to a more restricted region (−120°, 120°).

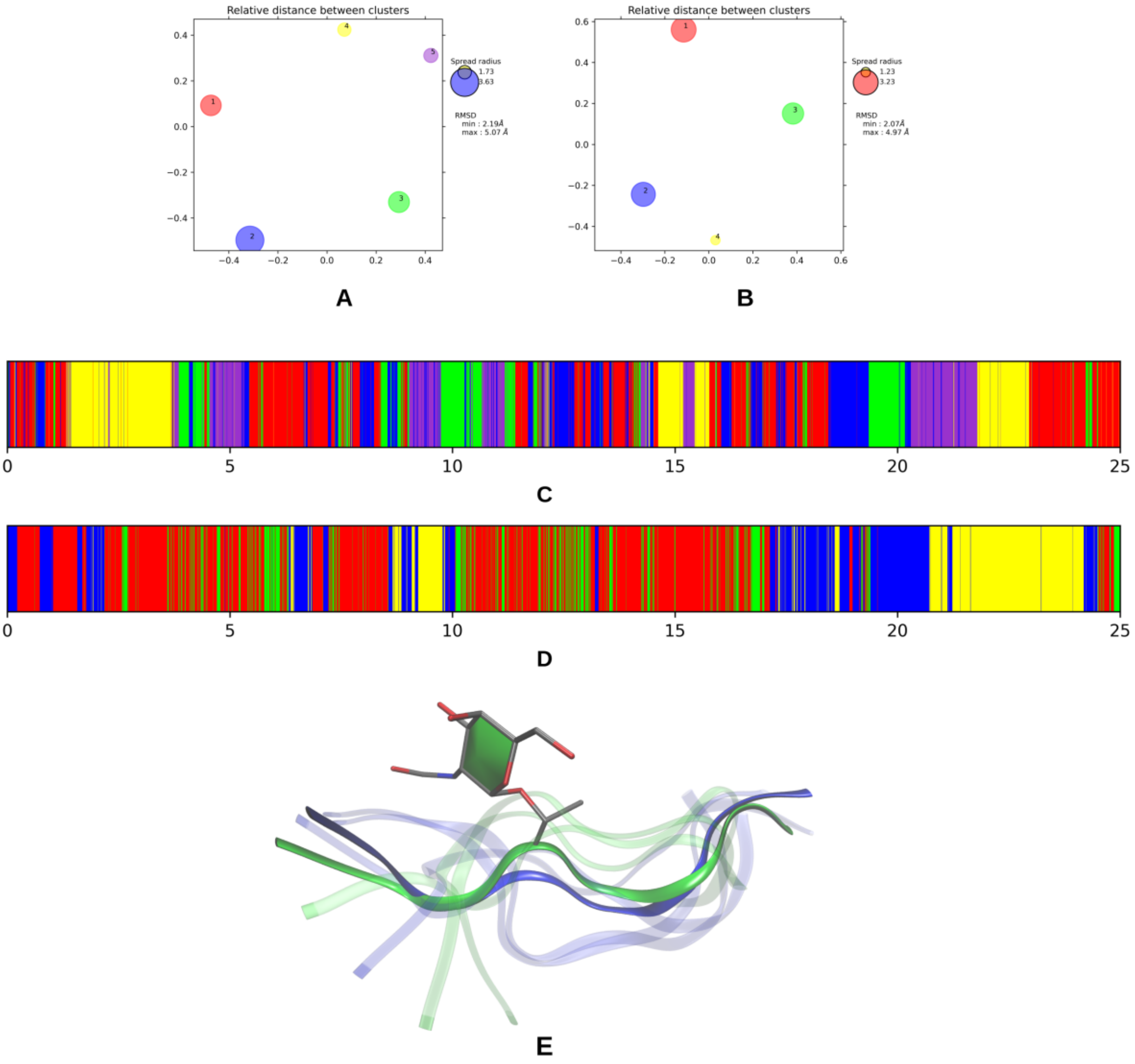

Figure S4: Results from cluster analysis using TTClust. Distance between clusters for antigen and Tn-antigen in solution (A, B). A linear projection of cluster by color over the frames analyzed for antigen and Tn-antigen in solution (C, D). Conformation of the clusters from TTClust (E). The antigen in blue with first cluster conformation in opaque blue. Tn-antigen in green with first cluster conformation in opaque green.

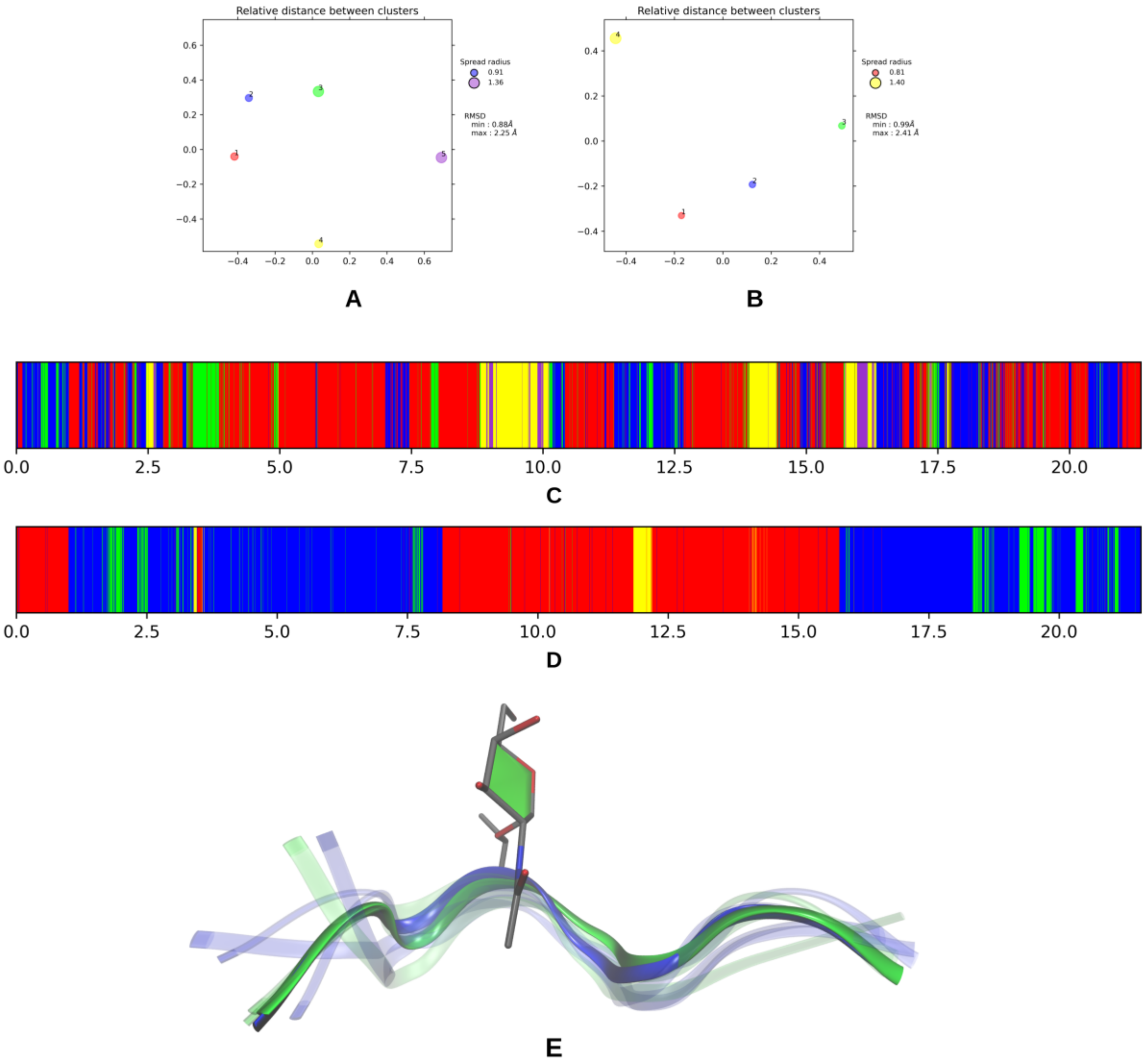

Figure S5: Results from cluster analysis using TTClust. Distance between clusters for antigen and Tn-antigen in antibody (A, B). A linear projection of cluster by color over the frames analyzed for antigen and Tn-antigen in antibody (C, D). Conformation of the clusters from TTClust (E). The antigen in blue with first cluster conformation in opaque blue. Tn-antigen in green with first cluster conformation in opaque green.

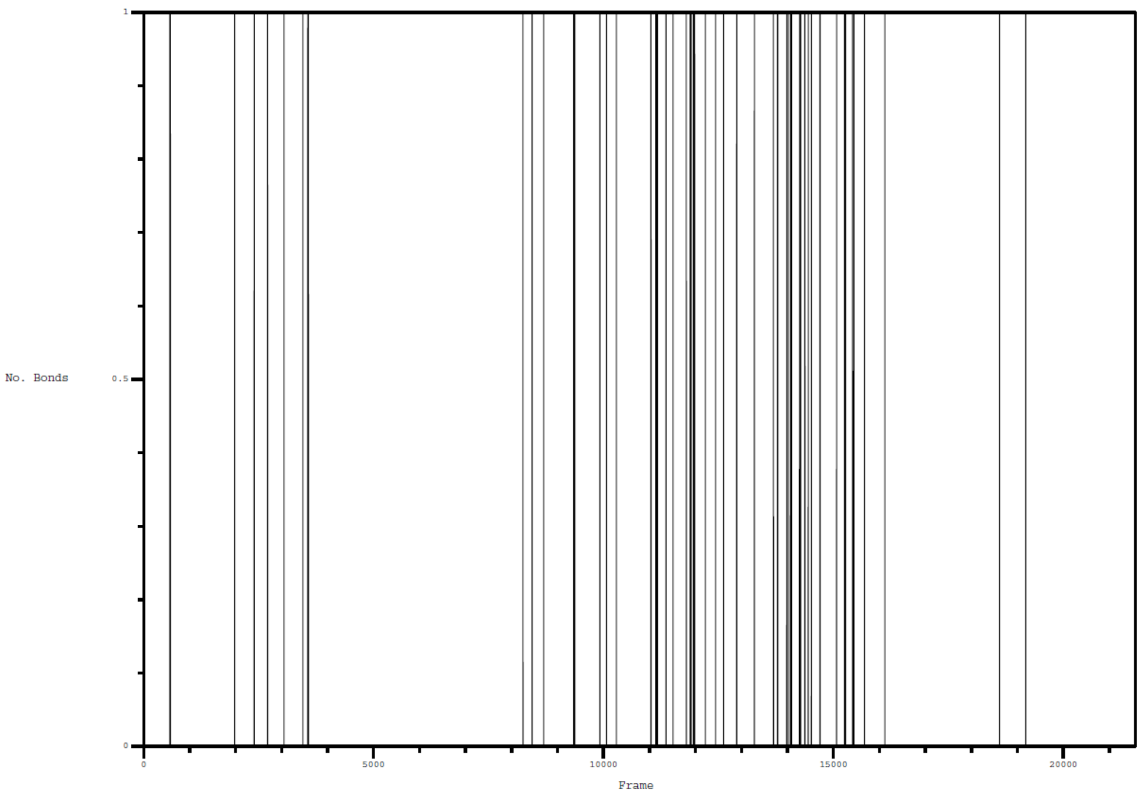

Figure S6: A plot of the hydrogen-bond occurrence between the hydroxy group of Tyr100 of chain B of the antibody and the 6-hydroxy group of the GalNAc. Both hydroxyl groups act as donors and acceptors, and this plot does not distinguish between them.

Hydrogen bonding

Tables S1–S7 contain hydrogen-bonding results from the hydrogen-bond analysis tool MDAnalysis. This tool conveniently summarizes the hydrogen-bond occupancy as a percentage but is not very specific about the hydrogen-bond contact (VMD Hydrogen-bond analysis was used for further analysis and some specifics are detailed in the main text). 'Main' represents the main chain (i.e., carbonyl and amino group), while 'Side' represents the side chain. For proline, which is cyclic, 'Side' refers to the amine group which has cyclized and viewed as part of the side chain. AGA1 is the sugar (GalNAc). AGA1Main and AGA1Side are not specific as there are multiple hydrogen-bonding-donor and acceptor sites in the sugar.

Table S1: Hydrogen-bonding interactions for the peptide antigen in solution.

donor	acceptor	occupancy
ARG5-Side	ASP3-Side	31.83%
ARG5-Side	PRO8-Main	14.32%
ARG5-Side	PRO8-Side	13.67%
ARG5-Main	ASP3-Side	2.76%
ARG5-Side	PRO6-Main	0.94%
ARG5-Side	THR4-Main	0.70%
ARG5-Side	ASP3-Main	0.52%
ARG5-Side	PRO2-Main	0.44%
ARG5-Side	ALA7-Main	0.26%
THR4-Side	ASP3-Main	0.23%
THR4-Main	ASP3-Side	0.10%
THR4-Side	ASP3-Side	0.08%
ALA1-Main	PRO8-Side	0.06%
THR4-Side	ARG5-Main	0.04%
ARG5-Side	ARG5-Main	0.04%
THR4-Main	ALA1-Main	0.04%
ALA1-Main	THR4-Side	0.02%
ALA7-Main	ARG5-Main	0.02%
THR4-Side	PRO2-Main	0.02%
ASP3-Main	ALA1-Main	0.01%
THR4-Side	ALA1-Main	0.01%
ARG5-Side	THR4-Side	0.01%
ALA1-Main	ASP3-Side	0.01%

THR4-Main	PRO2-Main	0.00%
ARG5-Side	ALA1-Main	0.00%

Table S2: Hydrogen-bonding interactions for the Tn-peptide antigen in solution.

**donor**	**acceptor**	**occupancy**
ARG5-Side	PRO8-Side	26.69%
ARG5-Side	PRO8-Main	26.58%
ARG5-Side	ASP3-Side	12.45%
ARG5-Side	PRO2-Main	7.13%
AGA1-Side	AGA1-Main	6.92%
AGA1-Main	ASP3-Main	1.37%
ARG5-Side	ALA7-Main	1.14%
AGA1-Side	PRO2-Main	0.54%
AGA1-Main	ARG5-Main	0.44%
AGA1-Side	ASP3-Main	0.38%
ARG5-Side	ASP3-Main	0.34%
ARG5-Side	AGA1-Side	0.22%
ARG5-Side	PRO6-Main	0.21%
ARG5-Main	AGA1-Side	0.19%
ARG5-Side	THR4-Main	0.12%
THR4-Main	ASP3-Side	0.10%
AGA1-Side	ASP3-Side	0.08%
AGA1-Side	AGA1-Side	0.08%
AGA1-Side	ARG5-Main	0.08%
AGA1-Main	THR4-Main	0.07%

ARG5-Main	AGA1-Main	0.06%
AGA1-Main	PRO2-Main	0.06%
ALA7-Main	AGA1-Side	0.02%
AGA1-Side	ALA1-Main	0.02%
ALA1-Main	AGA1-Side	0.02%
ALA7-Main	ARG5-Main	0.02%
AGA1-Main	ASP3-Side	0.01%
ARG5-Side	ARG5-Main	0.01%
ALA1-Main	THR4-Main	0.01%
THR4-Main	ALA1-Main	0.01%
AGA1-Side	PRO8-Main	0.01%
AGA1-Side	ALA7-Main	0.00%
ARG5-Side	AGA1-Main	0.00%

Table S3: Intramolecular hydrogen-bonding interactions for the peptide antigen in the antibody.

**donor**	**acceptor**	**occupancy**
ALA1-Main	THR4-Side	0.14%
THR4-Side	ARG5-Main	0.10%
THR4-Side	THR4-Main	0.01%
THR4-Main	ALA1-Main	0.01%

Table S4: Intramolecular hydrogen-bonding interactions for the Tn-peptide antigen in the antibody.

donor	acceptor	occupancy
AGA1-Side	AGA1-Main	29.08%
AGA1-Main	THR4-Main	23.04%
AGA1-Side	AGA1-Side	0.02%
AGA1-Main	ALA1-Main	0.02%
ALA7-Main	AGA1-Main	0.01%

Table S5: Hydrogen-bonding interactions between the peptide antigen and chain A of the AR20.5 antibody.

donor	acceptor	occupancy
ARG5-Side	GLU39-Side	141.21%
LYS58-Side	ASP3-Side	44.44%
TYR37-Side	PRO2-Main	42.55%
ARG55-Side	ASP3-Side	38.11%
TYR54-Side	ASP3-Side	28.51%
ARG55-Side	ALA1-Main	0.53%
TYR37-Side	ASP3-Main	0.15%
TRP101-Side	PRO6-Main	0.14%
TYR37-Side	THR4-Main	0.07%
ARG55-Side	PRO2-Main	0.05%

Table S6: Hydrogen-bonding interactions between the Tn-peptide antigen and chain A of the AR20.5 antibody.

donor	acceptor	occupancy
ARG5-Side	GLU39-Side	137.49%
TYR37-Side	PRO2-Main	46.73%
LYS58-Side	ASP3-Side	42.80%
ARG55-Side	ASP3-Side	37.77%
TYR54-Side	ASP3-Side	31.44%
ARG55-Side	ALA1-Main	0.66%
TYR37-Side	THR4-Main	0.32%
ARG55-Side	PRO2-Main	0.04%
TYR37-Side	ASP3-Main	0.00%

Table S7: Hydrogen-bonding interactions between the Tn-peptide antigen and chain B of the AR20.5 antibody.

donor	acceptor	occupancy
TYR100-side	AGA1-Side	0.15%
AGA1-Side	TYR100-side	0.13%
